# Efficacy of Internet-Based Self-Monitoring Interventions on Maternal and Neonatal Outcomes in Perinatal Diabetic Women: A Systematic Review and Meta-Analysis

**DOI:** 10.2196/jmir.6153

**Published:** 2016-08-15

**Authors:** Ying Lau, Tha Pyai Htun, Suei Nee Wong, Wai San Wilson Tam, Piyanee Klainin-Yobas

**Affiliations:** ^1^ National University of Singapore Alice Lee Centre for Nursing Studies Singapore Singapore; ^2^ National University of Singapore Medical Resource Team, National University of Singapore Libraries Singapore Singapore

**Keywords:** Internet, pregnancy in diabetics, interventions, meta-analysis

## Abstract

**Background:**

Self-monitoring using the Internet offers new opportunities to engage perinatal diabetic women in self-management to reduce maternal and neonatal complications.

**Objective:**

This review aims to synthesize the best available evidence to evaluate the efficacy of Internet-based self-monitoring interventions in improving maternal and neonatal outcomes among perinatal diabetic women.

**Methods:**

The review was conducted using Cochrane Central Register of Controlled Trials, PubMed, EMBASE, Cumulative Index to Nursing and Allied Health Literature, PsyINFO, Scopus, and ProQuest Dissertations and Theses to search for English-language research studies without any year limitation. A risk of bias table was used to assess methodological quality. Meta-analysis was performed with RevMan software. Cochran Q and *I*^2^ tests were used to assess heterogeneity. The overall effect was assessed using z tests at *P*<.05. Of the 438 studies identified through electronic searches and reference lists, nine experimental studies from 10 publications were selected.

**Results:**

Half of the selected studies showed low risk of bias and comprised 852 perinatal diabetic women in six countries. The meta-analysis revealed that Internet-based self-monitoring interventions significantly decreased the level of maternal glycated hemoglobin A1c (z=2.23, *P*=.03) compared to usual care among perinatal diabetic women at postintervention. Moreover, Internet-based self-monitoring interventions significantly decreased the cesarean delivery rate (z=2.23, *P*=.03) compared to usual care among the mixed group at postintervention.

**Conclusions:**

This review shows neonatal or other maternal outcomes are similar between Internet-based self-monitoring interventions and usual diabetes care among perinatal diabetic women. The long-term effects of the intervention must be confirmed in future studies using randomized controlled trials and follow-up data.

## Introduction

Diabetes mellitus (DM) is one of the most common complications of pregnancy; preexisting diabetes mellitus (type 1 or type 2) and gestational diabetes mellitus (GDM) affect approximately 2.5% to 2.7% and 4.6% to 8.0% of all pregnant women, respectively [1]. Both GDM and preexisting diabetes are associated with increased medical costs and perinatal morbidity [1]. Existing interventions must be improved considering the increasing global incidence of diabetic pregnancy with serious perinatal outcomes [2]. Self-monitoring intervention is important in reducing maternal and neonatal complications related to diabetic pregnancies, both in cases of preexisting diabetes [3] and GDM [4]. Self-monitoring refers to systematic observation and recording of ongoing goal-directed activities [5] based on self-regulation theory [6]. Self-regulation involves self-awareness of the current condition of an individual [7]. Awareness could trigger a self-evaluation response involving the interpretation of one’s condition against a goal or standard; after self-evaluation, a series of responses could be determined through self-adjustment and self-reinforcement [1,6]. Self-monitoring capitalizes on this motivation to achieve glycemic control [8], improve weight management [9], and reduce hospitalization and readmission rates [10].

Self-monitoring using the Internet offers new opportunities to engage participants in self-management. A previous study [11] suggested that self-monitoring using Internet-based interventions and face-to-face interventions elicited similar outcomes among the patients. Development of Internet-based interventions by using theory-based methods could promote substantial changes in the health behavior of a patient [12]. The Internet offers a diverse range of strategies for exchanging information and gaining knowledge [13] and thus can provide interactive ways to integrate communication with sensor-based systems (glucometer and pedometer) for transmitting information to a device or computer [14,15]. Sensors are used to record and transmit data to a computer, which then transmits the data to the provider and provides personalized/tailored feedback to the individual [14,15] regarding self-monitoring compliance with treatments and self-adjustment to diet, activity, and weight management.

Internet-based interventions employ a tracking system to improve self-reinforcement by using reminders (cues to action) [16], alerts [14], or graphic progress [17] through text messages (short message service, SMS) and email. Asynchronous and synchronous interactions generate identical interactional benefits [18]. Peer-support interactivity allows women to interact with one another with a pseudonym [15]; this process could empower women to take ownership of their well-being and initiate resolutions for issues they are encountering, thereby contributing to a sense of increased self-efficacy among perinatal diabetic women [19]. A longitudinal follow-up is important to test the sustainability of self-monitoring patterns over an extended period [20]. The advantages of using the Internet to deliver interventions include low cost, easy distribution, and convenient delivery to multiple locations at specific times [4,21]. Internet access is increasingly used as an educational and supportive source of information for perinatal women [22,23]. Internet-based interventions are rapidly developed with increased access to instant cyber connectivity; however, the effect of Internet-based self-monitoring on improving maternal and neonatal outcomes among perinatal diabetic women remains unclear.

Meta-analysis is used to document the application of Internet-based self-monitoring interventions among general diabetic population [24-26]. However, only a few studies were conducted on perinatal diabetic women. Four reviews focused on the use of technologies to evaluate healthy pregnant women in terms of maternal outcomes [27], women with complicated pregnancies in terms of cost effectiveness [28], a mixed group of patients (with type 1 DM and GDM) in terms of maternal-neonatal outcomes [29], and patients with GDM in terms of maternal outcomes [30]. These studies reported mixed results, did not include ongoing studies without outcomes [27], lacked systematic searching strategies [28,29], and evaluated limited studies (n=3) [30]. None of the studies focused on Internet-based self-monitoring approaches. Hence, further research must be performed, particularly in light of the rapid improvements in technologies worldwide. This review aims to systematically assess studies that examined Internet-based self-monitoring interventions for improving maternal and neonatal outcomes among perinatal diabetic women.

## Methods

This study was performed in accordance with the recommendations of the Preferred Reporting Items for Systematic Reviews and Meta-Analyses (PRISMA) statement [31]. The protocol is registered to the PROSPERO database (CRD42016034142).

### Eligibility Criteria

The full inclusion and exclusion criteria for the systematic review are described in Multimedia Appendix 1. Studies were included if they met the following criteria:

Population: perinatal women aged 18 years and older with GDM, type 1 DM, and/or type 2 DM;

Interventions: interact with perinatal diabetic women to undertake one or more of the following components associated with self-awareness, self-interpretation, self-adjustment, or self-reinforcement of glycemic level, physical activities, dietary intake, weight management, or medication adherence [7,10] by using the Internet;

Comparison: usual diabetes care as control group;

Outcomes: primary outcomes included glycated hemoglobin A_1c_ (HbA_1c_) level, cesarean delivery, neonatal birth weight, and neonatal hypoglycemia at postintervention; secondary outcomes included biological outcomes (fasting blood glucose, weight gain, and change in body mass index [BMI] or weight), cognitive outcomes (satisfaction rate, empowerment, self-efficacy, or health-related quality of life), behavioral outcomes (insulin treatment rate or compliance rate with self-monitoring), emotional outcomes (depression or stress), and neonatal outcomes (large for gestational age or macrosomia) at postintervention; and

Type of design: experimental studies that were either a randomized controlled trial (RCT) or controlled clinical trial (CCT). We excluded studies if they were nonexperimental, qualitative, protocol, or conference papers regarding general diabetic populations.

### Search Strategy

The search strategy aimed to find published or unpublished studies written in English. No restriction was applied to the search performed from inception until February 16, 2016 in the following electronic databases: Cochrane Central Register of Controlled Trials (CENTRAL), PubMed, EMBASE, Cumulative Index to Nursing and Allied Health Literature (CINAHL), PsycINFO, Scopus, and ProQuest Dissertations and Theses. Index and keyword terms were used (Multimedia Appendix 2). The keywords were exploded and truncated following the syntax rules of each database. Unpublished trials of relevance to the review were searched from the Clinical Trials Registry (www.clinicaltrials.gov). Unpublished data were requested if eligible trials maximized the scope of the search. Finally, we searched the reference lists of the included studies and relevant previous reviews to check for additional eligible studies.

### Study Selection

Two authors (LY and TP) independently screened the titles and abstracts of the identified references from the literature search to identify potentially eligible studies. The full texts of the remaining references were evaluated. Ineligible reports were excluded based on inclusion criteria, and the reasons for exclusion were recorded. A third reviewer (KY) resolved disagreements between the two reviewers regarding inclusion of a study.

### Quality Assessment

After identifying studies that fulfilled the selection criteria and verifying their eligibility by reading the completed articles, the studies were subjected to quality assessment. The quality of the studies was independently judged using criteria for determining bias in intervention studies recommended by the Cochrane Handbook for Systematic Reviews of Interventions [32]. The following indicators of internal validity specific to the methodology of RCT were collected: (1) random sequence generation (selection bias), (2) allocation concealment (selection bias), (3) blinding of participants and personnel (performance bias), (4) blinding of outcome assessment (detection bias), (5) incomplete outcome data (attrition bias), and (6) selective reporting (reporting bias) [32]. Assessment related to risk biases was assigned a judgment of “low risk,” “high risk,” or “unclear risk” of bias. One reviewer (LY) reviewed all studies with a subset reviewed by a second reviewer (TP). Disagreements were settled through discussion or consulting a third reviewer (KY).

### Data Extraction

Two of the authors (LY and TP) extracted relevant data from all included articles. The following variables were obtained using structured data extraction items based on setting, country, design, population, gestation, age, intervention, control, sample size, outcomes, attrition, and intention-to-treat (ITT) analysis. The details of self-monitoring interventions were extracted based on components (glycemic, diet, weight gain control, physical activities, or/and medication adherence), transmission (asynchronous or asynchronous), functionality, facilities, interactivity, provider, peer support, duration, and follow-up. The two authors (LY and TP) thoroughly reviewed the summary tables for accuracy and relevance. When relevant data were missing or questionable in both published and unpublished trials, the authors were contacted for verification and to obtain additional information. Among 59 full-text articles, 10 were not clear because they had insufficient details (n=2) or no (n=8) outcomes. Although 10 authors were approached, none responded to our queries. Therefore, we excluded these 10 studies in the review.

### Statistical Analysis

RevMan software (Review Manager version 5.3 for Windows from the Nordic Cochrane Center, the Cochrane Collaboration, 2014) was used for meta-synthesis. Risk ratio (RR) was used as the effect measure for dichotomous outcome with Mantel-Haenszel method. Mean difference was used for continuous outcomes with inverse-variance method. Heterogeneity between studies was evaluated using Cochran Q (chi-square test) and *I*^2^ statistics. The statistical significance for heterogeneity using the chi-square test was set as *P*<.10. The *I*^2^ statistic was applied to describe total variations in study estimates because of heterogeneity. Heterogeneity degree was estimated using *I*^2^ by setting 0%, 25%, 50%, or 75% for no, low, moderate, and high heterogeneity, respectively [33]. The fixed-effect model was used in cases without significant heterogeneity (*P*>.10), and the DerSimonian and Laird random-effects model was used in cases with heterogeneity among the studies (*P*<.10) and *I*^2^ values of more than 50% [33]. Subgroup analysis was performed to explore the source of heterogeneity, and the predefined subgroup was the type of DM.

## Results

**Figure 1:**
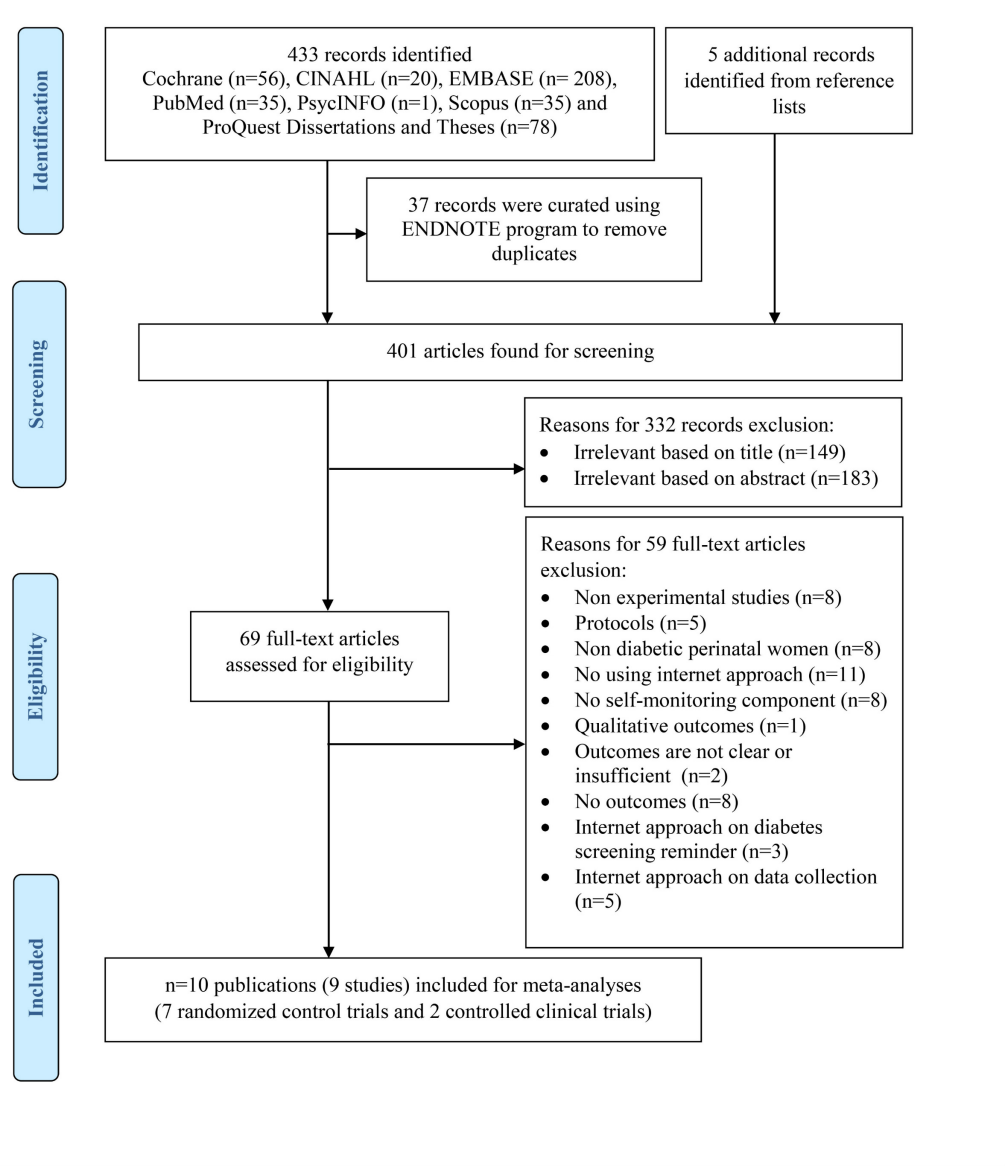
PRISMA flow diagram of article selection procedure.

Figure 1 shows the selection process (PRISMA flow diagram). A total of 438 studies were identified from the initial database search and reference lists. Of these studies, 37 articles were curated using Endnote to remove duplicates. Subsequently, 401 studies were included for screening and 332 articles were excluded based on analysis of text words in titles and abstracts. In all, 69 full-text articles were retrieved, reviewed, and selected based on relevance and quality for eligibility. Of these, 59 articles were excluded because of the following: nonexperimental nature; type of protocol; nondiabetic perinatal women as subject; not using Internet approach; lack of self-monitoring component; reported qualitative, unclear, insufficient, or no outcomes; and Internet approach employed on diabetes screening, reminder, data collection. Finally, nine studies from 10 publications were identified for inclusion in this systematic review.

### Study Characteristics

This meta-analysis included nine studies with 852 participants conducted across four countries (Table 1), which included the United States (n *=* 5) [15,16,34-36], Spain (n=2) [14,37,38], Italy (n=1) [39], and Ireland (n=1) [40]. All these studies were published articles. Research was conducted between 2007 [36] and 2015 [16]; 2015 had the highest number of publications (n=3) [14,16,40]. Seven of the studies used RCT designs and two used CCT designs [14,39]. The target populations were perinatal women with GDM or impaired glucose tolerance (n=5) [15,35,36], mixed group (n=4) [14,16,39], type 1 DM (n=0), and type 2 DM (n=0). The sample sizes varied among the nine studies and ranged from 19 [41] to 235 [39]. Nine studies reported more than one outcome. Attrition rates ranged from 2% [14,37,38] to 32% [16]. None of the studies used ITT analysis, and eight studies were supported by grants.

**Table 1:** Characteristics of the nine selected studies (10 publications).^a^

Author, year [ref]	Setting/Country	Design	Population/gestation/age, mean (SD)	Intervention	N	Duration (weeks)	Outcomes	Attrition rate, %
Bartholomew et al 2015 [16]^b^	Antenatal clinic in Hawaii, USA	RCT	GDM or type 2 DM; <30 w; 33.2 (5.4)	Mobile phone, Internet technology (CIT)	I: 50; C: 50	3	Fasting and 2-hour postprandial blood compliance rate with SMBG; satisfaction rate	I: 20; C: 32
Carral et al 2015 [14]^b^	GDM unit in Cadiz, Spain	CCT	GDM, type 1 or 2 DM; <30 w; 33.8 (4.6)	Web-based telemedicine system	I: 40; C: 64	—	HbA_1c_ (%); weight gain; cesarean delivery rate; insulin treatment rate; neonatal birth weight; large for gestational age; neonatal hypoglycemia	I: 5; C: 14
Dalfra et al 2009 [39]	12 Diabetes clinics in Italy	CCT	GDM or type 1 diabetes; <30 w; 33.8 (4.6)	Telemedicine with Glucobeep server	I: 105; C:130	10	HbA_1c_ (%); weight gain; cesarean delivery rate; insulin treatment rate; neonatal birth weight; macrosomia; SF36; CES-D; DSS; DHDS	Total: 15; I:—; C:—
Given et al 2015 [40]^b^	2 Diabetes clinics in Ireland	RCT	GDM or IGT; 24-28 w; I: 33.5 (4.2), C: 30.1 (5.5)	Web-based telemedicine system	I: 24; C: 26	12	HbA_1c_ (%); cesarean delivery rate; insulin treatment rate; neonatal birth weight; macrosomia; neonatal hypoglycemia; satisfaction rate	I: 12.5; C:15.4
Homko et al 2007 [36]^b^	Antenatal clinic or one of its satellites in Philadelphia, PA	RCT	GDM; <33 w; 18-45, I: 29.8 (6.6), C: 29.2 (6.7)	Internet-based telemedicine system using ITSMyHealthfile and Lassoweb data engine	I: 34; C: 25	—	HbA_1c_ (%); FBS (mg/dL); cesarean delivery rate; DES; neonatal birth weight; large for gestational age; neonatal hypoglycemia	I: 5.8; C:13.8
Homko et al 2012 [35]^b^	Antenatal clinics (2) in Philadelphia, PA	RCT	GDM; <33 w; 18-45, I: 30.3 (6.0), C: 30.0 (7.5)	Internet-based telemedicine system with automatic telephone option	I: 40; C: 40	—	FBS (mg/dL); cesarean delivery rate; neonatal birth weight; large for gestational age; neonatal hypoglycemia	I: 10; C: 5
Kim et al 2012 [15]^b^	University health system in Michigan	RCT	GDM within 3 years; >18 years (—)	Web-based pedometer program	I: 28; C: 21	13	Change in weight; change in BMI; change in self-efficacy for weight and activity	I: 9.5; C:17.9
Nicklas et al 2014 [34]^b^	Hospital in Boston, MA	RCT	GDM; postnatal; 18-45 (—)	Web-based lifestyle intervention	I: 36; C: 39	24-40	Change in weight; change in BMI	I: 8.3; C:10.3
Pérez-Ferre et al 2010a,b [37,38]^b^	Diabetes unit of a hospital in Madrid, Spain	RCT	GDM; <28 w; I: 33.3 (5.6), C: 34.2 (5.2)	Web-based telemedicine system	I: 50; C: 50	12	HbA_1c_ (%); weight; weight gain; cesarean delivery rate; neonatal birth weight; large for gestational age; neonatal hypoglycemia	I: 2.0; C: 4.0

^a^ All studies had a usual treatment control group and none used ITT. —: Information not mentioned in article; BMI: body mass index; C: control group; CCT: controlled clinical trial; CES-D: Center for Epidemiologic Studies Depression Scale; DES: Diabetes Empowerment Scale; DHDS: Diabetes Health Distress Scale; DSS: Diabetes-related Stress Scale; FBS: fasting blood sugar; GDM: gestational diabetes mellitus; HbA_1c_: glycated hemoglobin A_1c_; I: intervention group; IGT: impaired glucose tolerance; ITT: intention-to-treat analyses; OGTT: Oral Glucose Tolerance Test; RCT: randomized controlled trial; SF36: SF-36 Health Survey; SMBG: self-monitoring of blood glucose.

^b^ These studies had grant support.

### Study Quality

**Figure 2:**
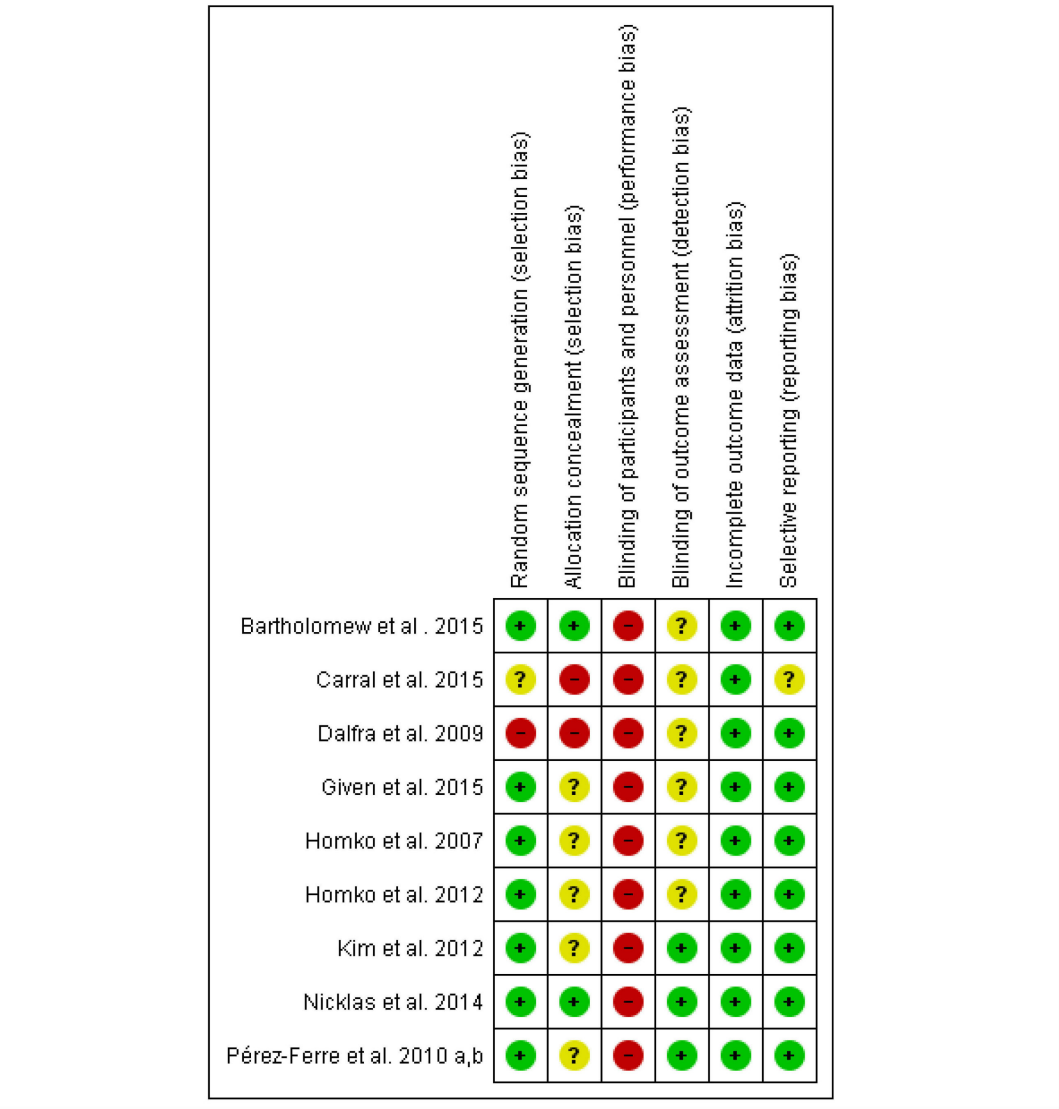
Risk of bias summary.

The summary of risk of bias is presented in Figure 2, and the risk of bias graph is shown in Multimedia Appendix 3. Seven of nine studies had adequate sequence generation for randomization. Two studies [16,34] had adequate allocation concealment. None of the studies implemented blinding of participants. Three studies [15,34,37,38] implemented blinding of outcome assessment. All studies addressed low-risk bias concerning incomplete outcome data. Eight had low-risk bias for selective reporting.

### Description of Internet-Based Self-Monitoring Interventions

Detailed elements of the Internet-based self-monitoring interventions are presented in Multimedia Appendix 4. The components of the self-monitoring interventions included glycemic control (n=8), diet control (n=7), physical activities (n=5), weight control (n=3) [15,34,39], and medication adherence (n=7). Functionalities of the interventions included system alert and reminder (n=4) [14,16,35,40], graphical progress (n=2) [15,37,38], and uploading, entering, and tracking own information (n *=* 3) [16,34,36] using website (n *=* 9), phone (n *=* 7), SMS text message (n *=* 5), email (n *=* 6), and animated video (n *=* 1) [34] that integrated communication with glucometer (n *=* 4) and pedometer (n *=* 1) [15]. The majority of the interventions used asynchronous communication (n=6), and three used synchronous communication [15,39,40] through two-way (n=9) feedback communication. The providers of the intervention were physicians (n *=* 7), nurses (n *=* 4), dietitians (n=1) [34], telemedicine service provider (n=1) [40], and study staff (n *=* 1) [15]. Only one intervention consisted of peer support using an online forum [15]. The duration of the intervention varied among the nine studies and ranged from 3 weeks [16] to 40 weeks [34]. Three of the studies [14,16,34] had follow-up after intervention. None of the studies reported using theoretical or conceptual framework to design their interventions.

### Efficacy of Internet-Based Self-Monitoring Interventions on Maternal Outcomes

**Figure 3:**
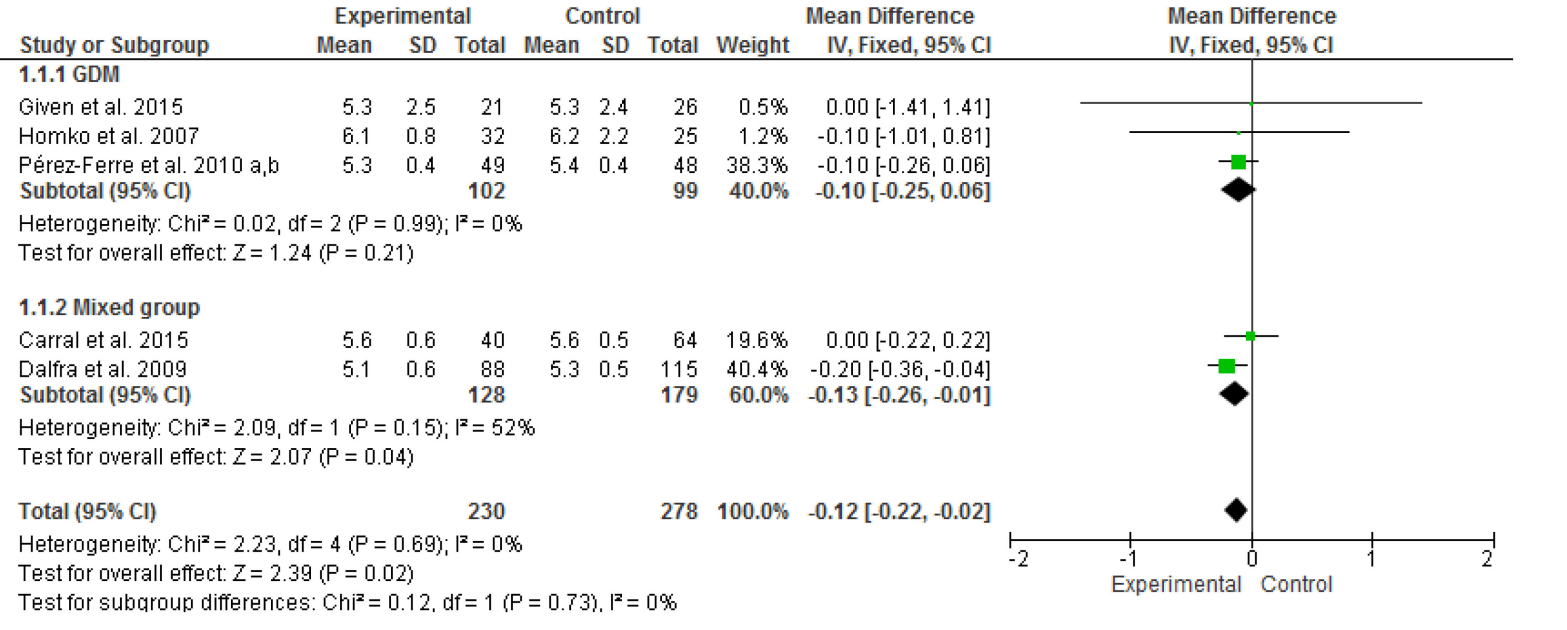
Forest plot of mean difference (95% CI) in change of HbA1c (%) for the Internet-based self-monitoring intervention and control groups. IV: inverse variance.

Five studies [14,36-39,40] assessed the efficacy of interventions among 508 perinatal women by using HbA_1c_ levels as the outcome. The meta-analysis revealed that the intervention significantly improved HbA_1c_ levels (mean difference −0.12, 95% CI −0.22 to −0.02), as determined using inverse-variance method and fixed-effects model (*I*^2^=0%, *P*=.69; Figure 3). A nonsignificant *P* value for the Cochran Q statistic indicated that the selected studies were homogeneous. The overall effect of intervention on HbA_1c_ was significant (*z*=2.39, *P*=.02). Subgroup analyses were performed to compare the effects of the interventions on HbA_1c_ between the GDM (n *=* 3) [36-38,40] and mixed groups (n *=* 2) [14,39]. However, no significant effect was found for subgroup differences (*P*=.73).

**Figure 4:**
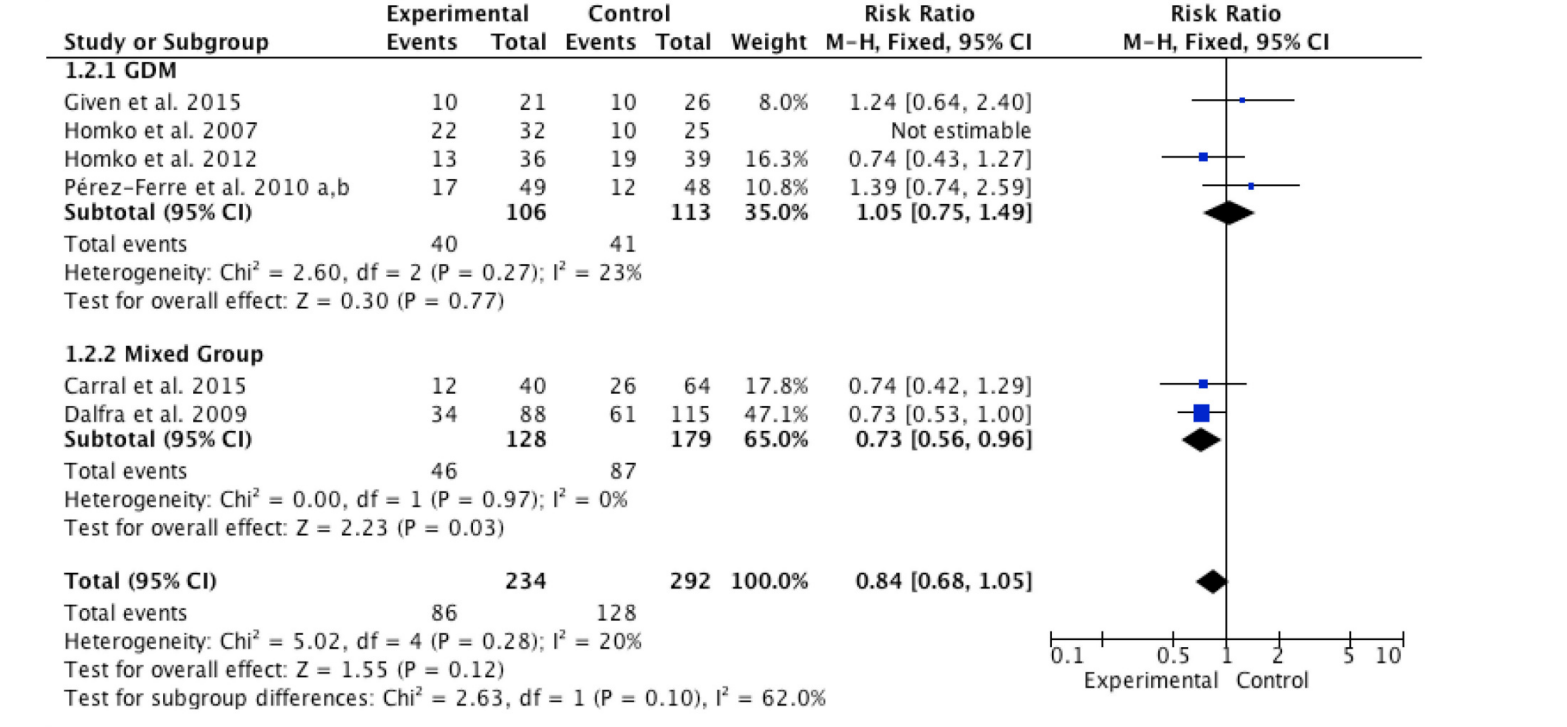
Forest plot of risk ratio in change of cesarean delivery rate for the Internet-based self-monitoring intervention and control groups.

Six studies [14,35-39,40] assessed cesarean delivery rate as outcomes of interventions among 526 perinatal women, and the meta-analysis showed low heterogeneity (*I*^2^*=* 20%, *P*=.28) (Figure 4). Moreover, the interventions did not significantly improve cesarean delivery rate for overall effect (RR=0.84, 95% CI 0.68-1.05; *z*=1.55, *P*=.12). Two subgroup analyses using the Mantel-Haenszel method and fixed-effects model revealed that the interventions significantly decreased the cesarean delivery rate among the mixed group (RR=0.73, *z*=2.23, *P*=.03) in two studies [14,39], but had no effect among the GDM group (RR=1.05, *z*=0.30, *P*=.77) in four studies [35-38,40]. No significant subgroup differences were found (*P*=.10). None and low heterogeneity were found between subgroups of women with GDM (*I*^2^*=* 0%, *P*=.97) and the mixed group (*I*^2^*=* 23%, *P*=.27).

### Efficacy of Internet-Based Self-Monitoring Interventions on Neonatal Outcomes

**Figure 5:**
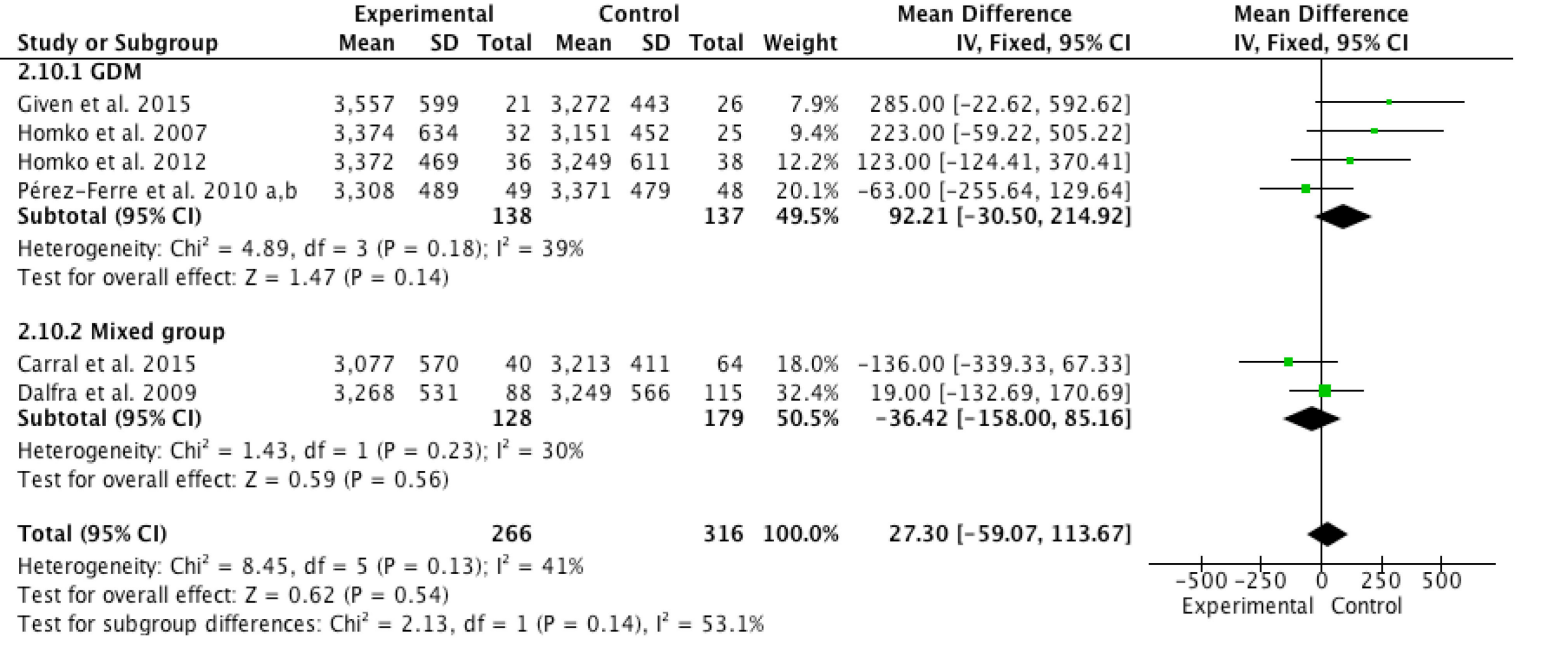
Forest plot of mean difference (95% CI) in change of neonatal body weight (grams) for the Internet-based self-monitoring intervention and control groups. IV: inverse variance.

Figure 5 shows the pooled meta-analysis results of six articles that determined the effect of interventions on neonatal body weight among 582 perinatal women. The meta-analysis showed low to moderate heterogeneity (*I*^2^*=* 41%, *P*=.13). Four studies [35-38,40] of GDM group and two studies [14,39] of mixed group revealed similar neonatal weight (mean difference=27.30, *z*=0.62, *P*=.54) between the Internet-based self-monitoring intervention and control groups. Two subgroup analyses were performed and no significant differences were found between intervention and control groups either in the GDM group (mean difference=92.21, *z*=1.47, *P*=.14) or the mixed group (mean difference=–36.42, *z*=0.59, *P*=.56). The heterogeneity of GDM group (*I*^2^=39%, *P*=0.18) and mixed group (*I*^2^=30%, *P*=.23) ranged from low to moderate. The test for subgroup differences was not significant (*P*=.14).

**Figure 6:**
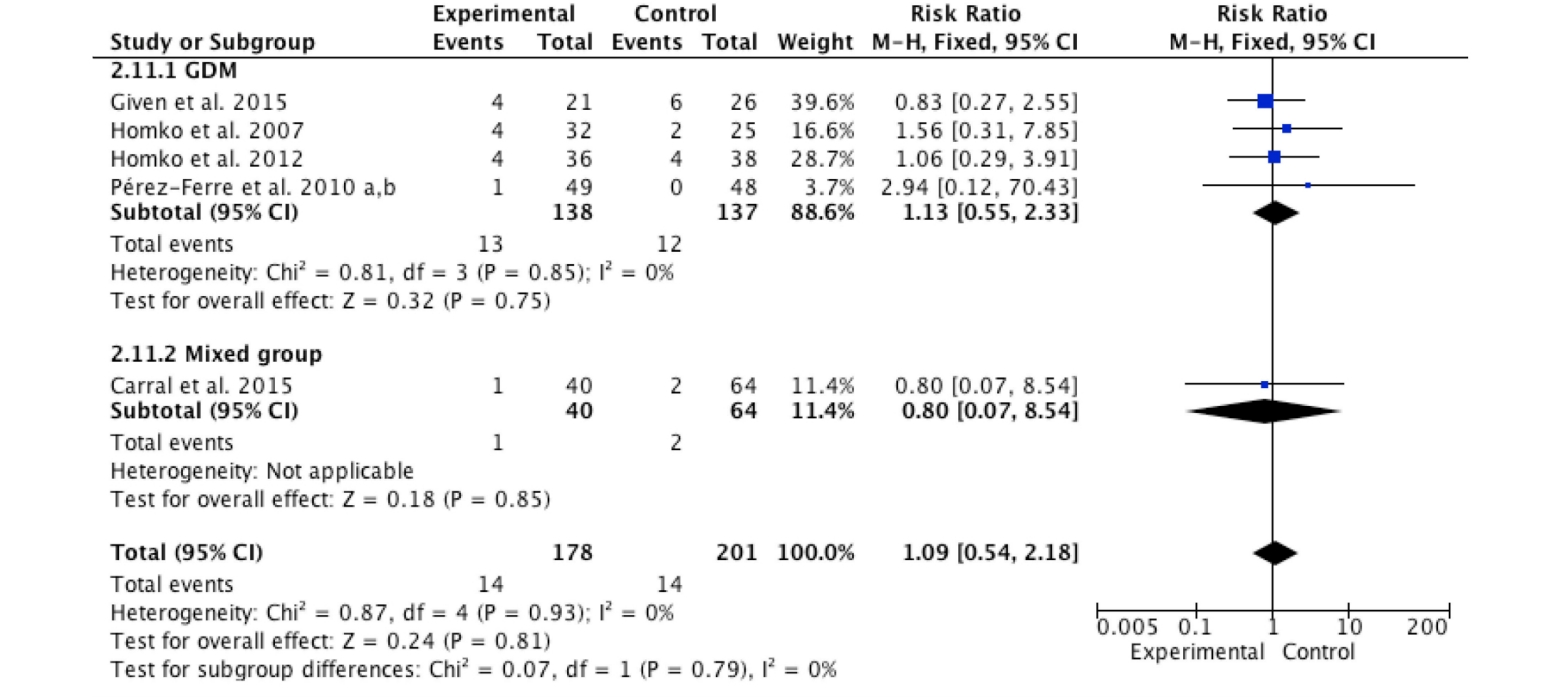
Forest plot of risk ratio for change in neonatal hypoglycemia rate for the Internet-based self-monitoring intervention and control groups.

Figure 6 shows the pooled meta-analysis results of five studies on neonatal hypoglycemia among 379 women. The intervention group demonstrated no significant difference on the overall effect (RR=1.09, *z*=0.24, *P*=.81) compared with the control group, as assessed using the Mantel-Haenszel method and fixed-effects model. No heterogeneity was found in the mixed group (*I*^2^*=* 0%, *P*=.85) and overall result (*I*^2^*=* 0%, *P*=.93). The result of subgroup analysis was not different (*P*=.79) between the mixed and GDM groups.

Table 2 summarizes the efficacy of the intervention on maternal outcomes including fasting blood sugar [35,36], weight gain [14,37,38], changes in BMI and weight [15,34], insulin treatment rate [14,37,38], satisfaction rate [16,40], compliance rate with self-monitoring of blood glucose [16], health-related quality of life [39], depressive symptoms [39], diabetic-related stress [39], diabetes health distress [39], diabetes empowerment [36], and change in self-efficacy for weight and activity [15], as well as neonatal outcomes including large for gestational age [14,35-38] and macrosomia [39,40]. The outcomes were not significantly different between intervention and control groups. Although the effects of diabetes-related stress and diabetes empowerment significantly differed in the Diabetes-related Stress Scale scores (*P=*.02) [39] and Diabetes Empowerment Scale scores (*P*=.003) [36], the findings of the single study could not contribute sufficient evidence to draw conclusions. The heterogeneity (*I*^2^) ranged from 0% in the pooled meta-analysis of three studies on weight gain [14,37,38] to 95% from the pooled meta-analysis of two studies on satisfaction rate [16,40] by using fixed- and random-effect models, respectively. Although we identified substantial heterogeneity (*I*^2^>50%), we encountered difficulty in investigating the result by using subgroup and sensitivity analyses for the two to three studies that indicated changes in BMI or weight [15,34], insulin treatment rate [14,37-39], and satisfaction rate [16,40].

**Table 2:** Efficacy of Internet-based self-monitoring interventions on other maternal and neonatal outcomes.^a^

Outcomes		Studies included, n	RR^b^/MD^c^ (95% CI)	Overall effect		Heterogeneity		Model
				*z*	*P*	*I* ^2^	*P*	
**Maternal outcomes**								
	Fasting blood sugar	2 [35,36]	–0.66^c^ (–4.28, 2.96)	0.36	.72	44%	.72	Fixed
	Weight gain	3 [14,37,38]	–0.48^c^ (–1.44, 0.47)	0.99	.32	0%	.98	Fixed
	Change in BMI	2 [15,34]	–0.91^c^ (–1.91, –0.09)	1.77	.08	64%	.09	Random
	Change in weight	2 [15,34]	–2.53^c^ (–5.10, –0.04)	1.93	.05	65%	.09	Random
	Insulin treatment rate	3 [14,37-39]	1.06^b^ (0.56, 2.02)	0.19	.85	71%	.03	Random
	Satisfaction rate	2 [16,40]	1.75^b^ (0.40, 7.58)	0.74	.46	95%	<.001	Random
	Compliance rate with self-monitoring of blood glucose	1 [16]	1.02^b^ (0.87, 1.20)	0.24	.81			
	SF-36 Physical component	1 [39]	–2.2^c^ (–4.50, 0.10)	1.88	.06	NA	NA	NA
	SF-36 Mental component	1 [39]	2.10^c^ (0.75, 4.95)	1.44	.15	NA	NA	NA
	CES-D	1 [39]	1.50^c^ (–1.35, 4.35)	1.03	.30	NA	NA	NA
	DSS	1 [39]	4.10^c^ (0.75, 7.45)	2.40	.02	NA	NA	NA
	DHDS	1 [39]	4.90^c^ (–0.20, 10.00)	1.88	.06	NA	NA	NA
	DES	1 [36]	0.40^c^ (0.14, 0.66)	3.00	.003	NA	NA	NA
	Change in self-efficacy for weight	1 [15]	2.79^c^ (–2.57, 8.15)	1.02	.31	NA	NA	NA
	Change in self-efficacy for activity	1 [15]	–1.40^c^ (–5.02, 2.22)	0.76	.45	NA	NA	NA
**Neonatal outcomes**								
	Large for gestational age	4 [14,35,36,37,38]	1.39^b^ (0.81, 2.40)	1.19	.23	0%	.68	Fixed
	Macrosomia	2 [39,40]	1.46^b^ (0.27, 7.98)	0.44	.66	69%	.07	Random

^a^ CES-D: The Center for Epidemiologic Studies Depression Scale; DES: Diabetes Empowerment Scale; DHDS, Diabetes Health Distress Scale; DSS, Diabetes-related Stress Scale; SF36: SF-36 Health Survey. NA: not applicable.

^b^ RR: risk ratio.

^c^ MD: mean difference.

## Discussion

This meta-analysis includes data from nine experimental studies, which included 852 women from four countries. The results revealed that the Internet-based self-monitoring interventions significantly decreased maternal HbA_1c_ levels compared with usual care among perinatal diabetic women at postintervention. Internet-based self-monitoring interventions significantly decreased the cesarean delivery rate compared to usual care among the mixed group at postintervention.

### Internet-Based Self-Monitoring Interventions

The major components of the interventions included self-monitoring glycemic control, medication adherence, physical activity, and diet control. Most of the interventions used websites, phone devices, and/or a glucometer through an asynchronous two-way feedback system. None of the selected studies developed interventions by using theoretical frameworks. Nevertheless, the hypothesized mechanism of action of the interventions should be described according to the Template for Intervention Description and Replication checklist and guide [42]. Theory can explain the rationale of the elements essential to the intervention and how the intervention really worked [43]. Theory can inform interventions in different ways, from identifying theoretical constructs to be targeted or mechanisms underlying particular behavior change techniques to selecting for women the approach that could most likely benefit them toward the right direction [12]. However, the sustainability of the positive findings from these studies is questionable because only three interventions [14,16,34] had follow-up mechanisms. Evidence demonstrated a gradual decline in adherence to self-monitoring of diet, exercise, medication adherence, and weight management [20]. Thus, future studies need to report the long-term effects of the intervention over an extended period. Only one study used a peer-support approach that provided diabetic women with opportunities to discuss problems with others experiencing the same issues [15]; this limitation suggests further research is warranted to determine whether peer-based online forums are effective in improving neonatal or maternal outcomes [19].

### Quality of the Evidence and Potential Biases

A high range of heterogeneity occurred between none (0%) to high (95%). The overall methodological quality of the studies included in the review was mixed and 78% (7/9) of the studies used methods to randomly assign women to either the intervention or the usual-care group using methods that we judged were at low risk of bias. This result was due to our selection criteria for either RCTs or CCTs. Thus, the majority prevented selection bias and insured against accidental bias. Only 22% (2/9) of the studies achieved adequate allocation concealment. Therefore, participants or providers could possibly foresee assignments to introduce selection bias. A potentially important source of bias in this meta-analysis was that none of the studies (0/9) achieved blinding of participants and personnel. Support intervention studies face considerable difficulties in blinding providers and women to an Internet-based group. Thus, all women would have performance bias. Only 33% (3/9) of the studies achieved an effective blinding of outcomes, perhaps owing primarily to the nature of the interventions. Even during an attempt made to blind outcome assessment, a high risk of response bias remained possible for outcomes relying on self-report and objective outcomes. Hence, the majority of women might harbor favorable expectation or increased apprehension in the Internet-based group or they might feel deprived or relieved in the usual-care group. The overall impact of sample attrition had a low-risk bias in all studies (9/9), which could improve the generalizability of findings and reduce attrition bias. Approximately 90% (8/9) of the studies reported primary and secondary outcomes that were reported in prespecified methods. Consequently, the selected studies did not obtain misleading results. None of the studies used ITT analysis, which is a method designed to solve problems of noncompliance and missing outcomes to maintain prognostic balance generated from the original random treatment allocation [44]. Therefore, all trials indicated overoptimistic estimates of the efficacy of the intervention on outcomes [44].

### Glycated Hemoglobin A1c

The results of this meta-analysis suggest that Internet-based self-monitoring interventions elicit significant effects on helping perinatal diabetic women to reduce their HbA_1c_ levels, which is consistent with the previous meta-analytic review among adults with type 2 DM [24,25]. A previous review identified 11 studies that analyzed HbA_1c_ levels and found that eight of these studies demonstrated a small significant decline in HbA_1c_ because of substantial heterogeneity (*I*^2^*=* 58%) in the effect interventions [24]. Although our review had no heterogeneity (*I*^2^*=* 0%) in the five identified studies, the small effect might be explained by different intensities of in-person contact between the intervention and control groups. We found the same in-person follow-up interval in both groups of two studies [36-38], but different intervals between the intervention and control group were indicated in three other studies [14,39,40]. A previous review [24] suggested that the intensity of in-person contact between consultation visits might relate to the efficacy of an Internet-based approach. We could not find the significant effect among subgroups of GDM [36-38,40] because of the small sample size, which had lower statistical power to select the true effect [45].

According to self-regulation theory [6], perinatal diabetic women could review their own data to obtain better understanding of their medical condition for self-awareness. The Internet could provide increased ease and convenience of self-monitoring because processing power and connectivity could allow remote access to information, and algorithms can target most of the components of existing face-to-face interventions [13]. Two-way personalized/tailored feedback with recommendations via email, online, or text message [14,36-39,40] helped gain diabetic knowledge and information for self-adjustment of glycemic control [14,36-39,40], diet control [14,36-39,40], appropriate activities control [36,39], weight gain control [39], and medication adherence control [14,36-39,40]. Sending automated alerts and reminders [14,40], voice messages [39], and visualizing data using graphs [37,38] encouraged engagement to the intervention to reinforce self-monitoring. Therefore, perinatal diabetic women capitalized on this motivation to improve HbA_1c_ levels.

### Cesarean Delivery Rate

Internet-based self-monitoring interventions were found to significantly decrease the cesarean delivery rate for a pool of 307 women in the mixed group [14,39], but no significant difference was found for a pool of 219 women with GDM [35-38,40]. The results of the meta-analysis are consistent with a previous meta-analytic review among women with GDM [30]. The study reported nonstatistically significant differences were found in cesarean delivery rates between telemedicine and a usual-care group; however, cesarean delivery rate analysis included only three studies [35,36,38]. This analysis includes an additional three studies [14,39,40]. The reason behind the significant decrease in the cesarean delivery rate in the mixed group but not in the GDM group remains unclear. Small sample size possibly underpowered the detection of any difference in cesarean delivery rate [45] among the GDM group, which suggests additional research is needed.

### Other Maternal and Neonatal Outcomes

This review showed similar neonatal or other maternal outcomes between the Internet-based self-monitoring interventions and usual diabetes care. However, the question remains as to whether Internet-based interventions may offer cost-effective service compared to usual care [28]. Interventions delivered over the Internet are likely to cost less than face-to-face services requiring frequent contact with health care personnel, and their relatively low delivery cost could result in an Internet-based intervention being more cost effective [4,26]. Currently, a dearth of evidence was detected regarding the effects of intervention on cognitive, behavioral, and emotional outcomes among perinatal diabetic women. Despite the identified nine individual cognitive, behavioral, and emotional outcomes in this review, evidence was too limited to draw any conclusion. Thus, additional good quality trials in this area are needed before firm conclusions can be made regarding the efficacy of Internet-based self-monitoring interventions on cognitive, behavioral, and emotional outcomes.

### Limitations

This review has several limitations. First, this review included only studies published in English, all of which were conducted in developed regions with high access to the Internet or mobile devices. Therefore, the results may not be applicable to marginalized groups in developing regions. Second, the subgroup analyses we performed prevented drawing definitive conclusions on the efficacy of Internet-based self-monitoring interventions. Subgroup analyses may pose significant interpretation problems, such as false positive or false negative outcomes [46,47]. The false positive outcomes were found for subgroup analyses when no true outcome exists, and have been estimated at 5% per subgroup [46,47]. The false negative outcomes were found because of the small number of outcome events in each subgroup. Therefore, limited statistical power minimized the random error among the estimates of event rates. Third, the small sample size is another limitation given that five of them used a small sample size from 49 [15] to 50 [40], and we found a lack of studies with type 1 or type 2 DM during pregnancy. Fourth, HbA_1c_ is known to be a 3-month mean measure of glycemic control, but the duration of intervention was not mentioned [14,36] or was less than 3 months [39] in three selected trials. Therefore, the validity of this measure as an outcome at postintervention might be questionable. Fifth, a nonsignificant effect was found in the GDM subgroup, but a significant effect was detected in the mixed group; thus, the effect of the type of diabetes rather than the true intervention effect was contentious. Finally, two studies [14,39] had CCT designs with insufficient control of extraneous variables, which diminished the internal validity of their findings.

### Implications for Future Research

Continuing research in this area is needed to develop effective Internet-based self-monitoring interventions to improve maternal and neonatal outcomes. Future studies should consider the theoretical base of the interventions [12] with a peer-support component [19] and long-term follow-up [20] to improve the efficacy and sustainability using a RCT design with ITT analyses [44]. However, determining the effective elements of Internet-based application is necessary. Further investigations are needed to divide these applications into specific components, features, transmission, functionality, facilities, interactivity, duration, and mode of delivery to differentiate the distinct effects of different functions [12]. This requirement is especially true in view of the lack of current research that explores the mechanism of effective interventions in different types of perinatal DM.

### Clinical Implications

Internet-based self-monitoring interventions may function as important extensions of the range of services to enhance the access of diabetic women to support with self-monitoring especially between consultation visits. Based on the findings of this study, websites that integrate communication with sensor-based systems and a tracing system should be considered high priority in designing self-monitoring interventions to improve maternal glycemic control and cesarean delivery rates. The ubiquity of the Internet facilitates dissemination of information and support to a broader audience and allows information and support to be tailored according to individual characteristics and experiences [26]. Perinatal diabetic women could access and review content at any time and place. Multimedia features and interactivity could accommodate different learning styles [48]. Data visualization capabilities and cloud computing offer accessible display of outcome information, flexible dissemination channels within and between service settings, and ready access to collaborative communication and shared resources for perinatal women and health care providers [13]. Furthermore, gaming technology, Bluetooth technology, interactive voice response, virtual reality, Facebook presence, as well as blogs and Global Positioning System navigation systems are another advancing wave of technological development that might potentially help map out new avenues to promote and support Internet-based self-monitoring among perinatal diabetic women.

### Conclusion

The rising popularity of the Internet might result in a shift from the traditional model of care toward an Internet-based health model. Internet-based self-monitoring interventions may introduce new approaches of improving maternal HbA_1c_ and cesarean delivery rates to perinatal diabetic women. Despite the limitations of this review and analysis, our findings have identified a need for future research to employ RCT designs with follow-up data to confirm the long-term effects of Internet-based self-monitoring interventions on maternal and neonatal outcomes among perinatal diabetic women.

## Appendix

Multimedia Appendix 1 Selection criteria for systematic review.

Multimedia Appendix 2 Index and keyword terms for searching in seven databases.

Multimedia Appendix 3 Risk of bias graph.

Multimedia Appendix 4 Description of Internet-based self-monitoring interventions in 9 selected studies.

## Abbreviations

BMI: body mass index
CCT: controlled clinical trial
CINAHL: Cumulative Index to Nursing and Allied Health Literature
DM: diabetes mellitus
GDM: gestational diabetes mellitus
HbA1c: glycated hemoglobin A1c
ITT: intention-to-treat
PRISMA: Preferred Reporting Items for Systematic Reviews and Meta-analysis
RCT: randomized controlled trial
RR: risk ratio
SMS: short message service
