# Supplementary material for: Efficacy of Internet-Based Self-Monitoring Interventions on Maternal and Neonatal Outcomes in Perinatal Diabetic Women: A Systematic Review and Meta-Analysis
Source: J Med Internet Res. 2016 Aug 15;18(8):e220. doi: 10.2196/jmir.6153 (PMC5004058; doi:10.2196/jmir.6153)
Supplement: Multimedia Appendix 1 [file jmir_v18i8e220_app1.pdf]

Multimedia Appendix 1

Selection criteria for the systematic review.

Criteria	Inclusion	Exclusion
Population	Perinatal women with<br>- Gestational diabetes mellitus,<br>- Type 1 diabetes mellitus or/and<br>- Type 2 diabetes mellitus	General population with<br>- Type 1 diabetes mellitus or/and<br>- Type 2 diabetes mellitus
Intervention	Technology-support self-monitoring intervention<br>- Glycemic control,<br>- Diet control,<br>- Appropriate physical activity,<br>- Weight management or/and<br>- Medication adherence	- Diabetic screening reminder system<br>- Not focused on the application of technology<br>- Technology-support for assessment
Comparison	- Standard diabetes care	Other diabetic interventions / programme
Primary outcomes	Maternal outcomes:<br>- Hemoglobin (Hb)A1c or/and<br>- Caesarean section rate<br>Neonatal outcomes:<br>- Neonatal birth weight or/and<br>- Neonatal hypoglycemia	
Secondary outcomes	Biological outcomes:<br>- Fasting blood glucose,<br>- Weight gain,<br>- Change in body mass index (BMI) or/and<br>- Change in weight,<br>Cognitive outcomes:<br>- Satisfaction rate,<br>- Empowerment,<br>- Change in self-efficacy for weight,<br>- Change in self-efficacy for activities or/and<br>- Health-related quality of life<br>Behavioral outcomes:<br>- Insulin treatment rate or/and<br>- Compliance rate with self-monitoring of blood glucose<br>Emotional outcomes:<br>- Depression or/and<br>- Stress<br>Neonatal outcomes:<br>- Large for gestational age<br>- Macrosomia	
Types of design	- Randomized controlled trial (RCT)<br>- Controlled clinical trial (CCT)	- Non-experimental studies<br>- Qualitative studies<br>- Review papers
Years of publication	No limit	No limit
Publication type	- Published primary research articles<br>- Unpublished theses	- Conference proceedings,<br>- Abstract only<br>- Book chapters review<br>- Letters<br>- Editorials
Language	English	Non-English
