# Supplementary material for: Efficacy of Internet-Based Self-Monitoring Interventions on Maternal and Neonatal Outcomes in Perinatal Diabetic Women: A Systematic Review and Meta-Analysis
Source: J Med Internet Res. 2016 Aug 15;18(8):e220. doi: 10.2196/jmir.6153 (PMC5004058; doi:10.2196/jmir.6153)
Supplement: Multimedia Appendix 2 [file jmir_v18i8e220_app2.pdf]

Multimedia Appendix 2

Indexed and keyword terms for searching in seven databases.

Database	Indexed and keyword terms
Pubmed	Search (((((("Cell phones"[Mesh] or "Computer Communication Networks"[Mesh] or "Computer systems"[Mesh] or "Computer-Assisted Instruction"[Mesh] or "Computers"[Mesh] or "Electronic mail"[Mesh] or "Hypermedia"[Mesh] or "Internet"[Mesh] or "Mobile Applications"[Mesh] or "Multimedia"[Mesh] or "Remote consultation"[Mesh] or "Telemedicine"[Mesh] or "Telephone"[Mesh] or "Therapy, computer-assisted"[Mesh] or "User-computer interface"[Mesh])))) OR (((((((cell phone* or cellular phone* or cellular telephone* or mobile phone* or smart phone*)) OR (computer-assisted or computer-based or web-based)) OR (Telemedicine or communication network or remote consultation or ((Electronic or mobile) and health) or telehealth or ehealth or m-health)) OR (Hypermedia or multimedia or multi-media or computer or internet or online or on-line)) OR (((world and wide and web) or (worldwide and web) or website*) or (electronic and health*)))))) AND ((((("Pregnancy in Diabetics"[Mesh] OR "Diabetes, Gestational"[Mesh])))) OR (((Gdm OR Gestational diabetes OR "Pregnancy in diabetes")))) Filters: Clinical Trial; Randomized Controlled Trial; Clinical Trial, Phase IV; Clinical Trial, Phase I; Clinical Trial, Phase II; Clinical Trial, Phase III; English; Adult: 19-44 years
Embase	'pregnancy diabetes mellitus'/exp OR 'pregnancy diabetes mellitus' OR 'gestational diabetes' OR 'pregnancy in diabetes' AND ('mobile phone'/exp OR 'mobile phone' OR 'computer network'/exp OR 'computer network' OR 'computer system'/exp OR 'computer system' OR 'computer assisted therapy'/exp OR 'computer assisted therapy' OR 'computer'/exp OR 'computer' OR 'hypermedia'/exp OR 'hypermedia' OR 'internet'/exp OR 'internet' OR 'multimedia'/exp OR 'multimedia' OR 'teleconsultation'/exp OR 'teleconsultation' OR 'telemedicine'/exp OR 'telemedicine' OR 'telephone'/exp OR 'telephone' OR (cell* OR mobile OR smart AND (phone* OR telephone*)) OR ('computer-assisted' OR 'computer assisted' OR 'computer-based' OR 'web-based') NEXT/6 (therap* OR treatment* OR education* OR instruction*) OR telemedicine OR 'communication networks' OR 'remote consultation' OR 'telehealth' OR 'electronic health' OR 'e-health' OR 'm-health' OR 'm health' OR 'mobile health' OR hypermedia OR multimedia OR 'multi media' OR computer* OR internet OR online OR 'on line' OR (world AND wide AND web) OR (worldwide AND web) OR website* OR (electronic AND health*)) AND ('clinical trial'/de OR 'controlled clinical trial'/de OR 'randomized controlled trial'/de OR 'randomized controlled trial (topic)'/de) AND ([adolescent]/lim OR [adult]/lim OR [aged]/lim OR [middle aged]/lim OR [young adult]/lim)
Scopus	(TITLE-ABS-KEY ("Pregnancy in Diabet*" OR "Diabetes Gestational" OR gdm OR "Gestational diabetes")) AND (TITLE-ABS-KEY (phone* OR telephone* OR computer OR ("computer assisted" OR "computer based" OR "web based") W/3 (therap* OR treatment* OR education* OR instruction*) OR (computer W/5 communicat* OR system) OR telemedicine OR "communication network" OR "remote consultation" OR telehealth OR ehealth OR mhealth OR computer OR "Electronic mail" OR hypermedia OR internet OR "Mobile App*" OR multimedia OR "User-computer interface" OR online OR worldwideweb OR WEBSITE))) AND ((TITLE-ABS-KEY ("clinical trials" OR "treatment outcome clinical trial" OR ((randomi?ed W/7 trial*) OR ((single OR doubl* OR tripl* OR treb*) AND (blind* OR mask*)) OR (controlled W/3 trial*)
Cochrane	#1 MeSH descriptor: [Cell Phones] explode all trees<br>#2 MeSH descriptor: [Computer Communication Networks] explode all trees<br>#3 MeSH descriptor: [Computer Systems] explode all trees<br>#4 MeSH descriptor: [Computer-Assisted Instruction] explode all trees<br>#5 MeSH descriptor: [Computers] explode all trees<br>#6 MeSH descriptor: [Electronic Mail] explode all trees<br>#7 MeSH descriptor: [Hypermedia] explode all trees<br>#8 MeSH descriptor: [Internet] explode all trees<br>#9 MeSH descriptor: [Mobile Applications] explode all trees<br>#10 MeSH descriptor: [Multimedia] explode all trees

Multimedia Appendix 2 (continued)

Indexed and keyword terms for searching in seven databases.

Database		Indexed and keyword terms
	#11	MeSH descriptor: [Remote Consultation] explode all trees
	#12	MeSH descriptor: [Telemedicine] explode all trees
	#13	MeSH descriptor: [Telephone] explode all trees
	#14	MeSH descriptor: [Therapy, Computer-Assisted] explode all trees
	#15	MeSH descriptor: [User-Computer Interface] explode all trees
	#16	#1 or #2 or #3 or #4 or #5 or #6 or #7 or #8 or #9 or #10 or #11 or #12 or #13 or #14 or #15
	#17	cell phone* or cellular phone* or cellular telephone* or mobile phone* or smart phone* or computer-assisted or computer-based or web-based or Telemedicine or communication network or remote consultation or ((Electronic or mobile) and health) or telehealth or ehealth or m-health or Hypermedia or multimedia or multi-media or computer or internet or online or on-line or (world and wide and web) or (worldwide and web) or website* or (electronic and health*)
	#18	MeSH descriptor: [Pregnancy in Diabetics] explode all trees
	#19	MeSH descriptor: [Diabetes, Gestational] explode all trees
	#20	#18 or #19
	#21	Gdm or Gestational diabetes or "Pregnancy in diabetes"
	#22	#20 or #21
	#23	#16 or #17
	#24	#22 and #23
CINAHL	S1	(MH "Diabetes Mellitus, Gestational") OR "Diabetes mellitus, gestational" OR (MH "Pregnancy in Diabetes+")
	S2	(MH "Cellular Phone+") OR "Cellular Phone" OR (MH "Smartphone+") OR (MH "Telephone+")
	S3	(MH "Computer Communication Networks+") OR (MH "Wireless Communications") OR "Computer communication network"
	S4	(MH "Computer Systems+") OR "Computer systems"
	S5	(MH "Computer Assisted Instruction") OR "Computer assisted instruction" OR (MH "Therapy, Computer Assisted+")
	S6	(MH "Computers, Portable") OR "Computer Portable"
	S7	(MH "Remote Consultation") OR "Remote consultation"
	S8	(MH "Internet+") OR "internet"
	S9	(MH "Telemedicine") OR (MH "Telehealth")
	S10	(MH "User-Computer Interface+") OR "User-Computer Interface"
	S11	(MH "Mobile Applications") OR "Mobile Applications"
	S12	(MH "Electronic Mail") OR "electronic mail"
	S13	S2 OR S3 OR S4 OR S5 OR S6 OR S7 OR S8 OR S9 OR S10 OR S11 OR S12
	S14	cell phone* or cellular phone* or cellular telephone* or mobile phone* or smart phone* or computer-assisted or computer-based or web-based or Telemedicine or communication network or remote consultation or ((Electronic or mobile) and health) or telehealth or ehealth or m-health or Hypermedia or multimedia or multi-media or computer or internet or online or on-line or ((world and wide and web) or (worldwide and web) or website*) or (electronic and health*)
	S15	S13 OR S14
	S16	gdm or "Gestational diabetes" or “Pregnancy in diabetes”
	S17	S1 OR S16
	S18	S15 AND S17
Psycinfo	1	Cell phone.mp. or exp Cellular Phones/
	2	Cellular Phones.mp. or exp Cellular Phones/
	3	exp Electronic Communication/ or Electronic Communication.mp.

Multimedia Appendix 2 (continued)

Indexed and keyword terms for searching in seven databases.

Database		Indexed and keyword terms
	4	exp Telecommunications Media/ or Telecommunications.mp.
	5	exp Computers/ or Computers.mp.
	6	exp Electronic Communication/ or exp Computer Mediated Communication/ or exp Telecommunications Media/ or Computer communication networks.mp.
	7	Internet.mp. or exp Internet/
	8	Information Systems.mp. or exp Information Systems/
	9	Communication Systems.mp. or exp Communication Systems/
	10	Computer Applications.mp. or exp Computer Applications/
	11	Computer-Assisted Instruction.mp. or exp Computer Assisted Instruction/
	12	Hypermedia.mp. or exp Hypermedia/
	13	Multimedia.mp. or exp Multimedia/
	14	Telemedicine.mp. or exp Telemedicine/
	15	Computer Assisted Therapy.mp. or exp Computer Assisted Therapy/
	16	exp Virtual Reality/ or Computer-assisted Virtual Reality.mp.
	17	Human Computer Interaction.mp. or exp Human Computer Interaction/
	18	Websites.mp. or exp Websites/
	19	exp Mobile Devices/ or Mobile Device.mp. or exp Human Computer Interaction/
	20	1 or 2 or 3 or 4 or 5 or 6 or 7 or 8 or 9 or 10 or 11 or 12 or 13 or 14 or 15 or 16 or 17 or 18 or 19
	21	(cell phone* or cellular phone* or cellular telephone* or mobile phone* or smart phone* or computer-assisted or computer-based or web-based or Telemedicine or communication network or remote consultation or ((Electronic or mobile) and health) or telehealth or ehealth or m-health or Hypermedia or multimedia or multi-media or computer or internet or online or on-line or ((world and wide and web) or (worldwide and web) or website*) or (electronic and health*)).mp. [mp=title, abstract, heading word, table of contents, key concepts, original title, tests & measures]
	22	('gdm' or 'gestational diabetes' or 'pregnancy in diabetes').mp. [mp=title, abstract, heading word, table of contents, key concepts, original title, tests & measures]
	23	20 or 21
	24	exp Pregnancy/ and exp Diabetes Mellitus/
	25	22 or 24
Proquest	S1	MESH#("Diabetes, Gestational" or "Pregnancy in Diabetics")
	S2	MESH#("Cell phones" or "Computer Communication Networks" or "Computer systems" or "Computer-Assisted Instruction" or "Computers" or "Electronic mail" or "Hypermedia" or "Internet" or "Mobile Applications" or "Multimedia" or "Remote consultation" or "Telemedicine" or "Telephone" or "Therapy, computer-assisted" or "User-computer interface")
	S3	Gdm OR Gestational diabetes OR “Pregnancy in diabetes”
	S4	cell phone* OR cellular phone* OR cellular telephone* OR mobile phone* OR smart phone* OR computer-assisted OR computer-based OR web-based OR Telemedicine OR communication network OR remote consultation OR ((Electronic OR mobile) AND health) OR telhealth OR health OR health OR Hypermedia OR multimedia OR multimedia OR computer OR internet OR online OR online OR (world AND wide AND web) OR (worldwide AND web) OR website* OR (electronic AND health*)
	S5	(S1 OR S3) AND (S2 OR S4)
	S6	DTYPE(clinical trial)
	S7	S5 AND S6
