# Supplementary figures and images for: Efficacy of Internet-Based Self-Monitoring Interventions on Maternal and Neonatal Outcomes in Perinatal Diabetic Women: A Systematic Review and Meta-Analysis
Source: J Med Internet Res. 2016 Aug 15;18(8):e220. doi: 10.2196/jmir.6153 (PMC5004058; doi:10.2196/jmir.6153)

### Multimedia Appendix 3

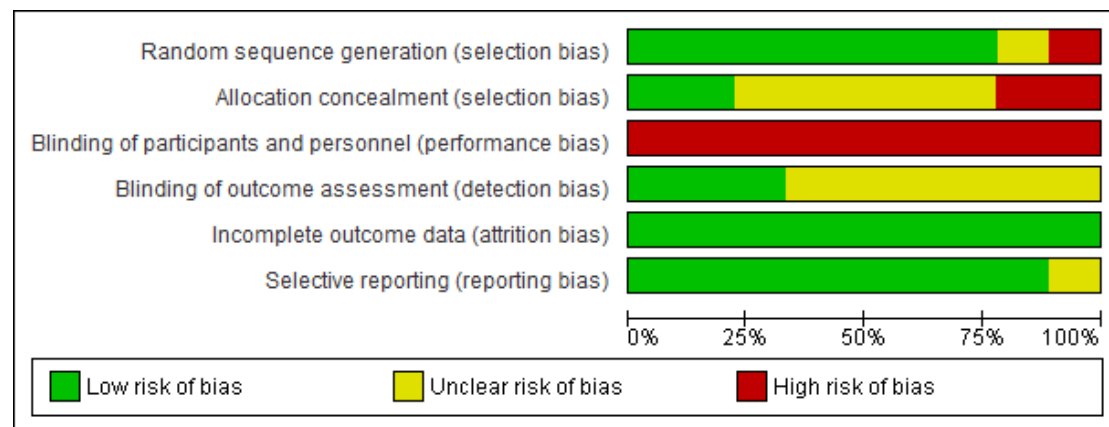

Risk of bias graph.

Supplement: Multimedia Appendix 3 [file jmir_v18i8e220_app3.pdf]
