# Supplementary material for: Efficacy of Internet-Based Self-Monitoring Interventions on Maternal and Neonatal Outcomes in Perinatal Diabetic Women: A Systematic Review and Meta-Analysis
Source: J Med Internet Res. 2016 Aug 15;18(8):e220. doi: 10.2196/jmir.6153 (PMC5004058; doi:10.2196/jmir.6153)
Supplement: Multimedia Appendix 4 [file jmir_v18i8e220_app4.pdf]

Multimedia Appendix 4

Description of Internet-based self-monitoring interventions in 9 selected studies.

Author, year [Reference]	Components of self-monitoring intervention					Functionality	Facilities	Transmission / Interactivity	Provider / Peer support	Theory	Follow-up
	Glycaemic	Diet	Activity	Weight	Adherence						
Bartholo-mew *et al.* 2015 (16)	Y	N	N	N	N	Upload blood glucose results Reminder via SMS	Website Phone SMS Glucometer	Asynchronous /Two-way Feedback	Nurses Physicians / No	No	3W
Carral *et al.* 2015(14)	Y	Y	N	N	Y	System alert via email	Website Email SMS Glucometer	Asynchronous /Two-way Feedback	Nurses Physicians / No	No	6-12 W Post-natal
Dalfra *et al.* 2009 (39)	Y	Y	Y	Y	Y	Voice message	Website Phone SMS Glucometer	Synchronous /Two-way Feedback	Physicians / No	No	No
Given *et al.* 2015 (40)	Y	Y	N	N	Y	System alert (Telemedici ne hub)	Website Phone Email	Synchronous /Two-way Feedback	Tele-medicine service provider / No	No	No
Homko *et al.* 2007 (36)	Y	Y	Y	N	Y	Daily enter blood glucose, diet and exercise and self-review the progress	Website Email	Asynchronous /Two-way Feedback	Nurses Physicians / No	No	No
Homko *et al.* 2012 (35)	Y	Y	Y	N	Y	Website Reminder (interactive voice response) Phone	Website Phone Email	Asynchronous /Two-way Feedback	Nurses Physicians / No	No	No
Kim *et al.* 2012 (15)	N	N	Y	Y	N	Graphically progress	Website Phone Email SMS Pedometer	Synchronous /Two-way Feedback	Study staff / Online forum	No	No
Nicklas *et al.* 2014 (34)	Y	Y	Y	Y	Y	Tracking of dietary intake and physical	Website Animated videos Phone	Asynchronous /Two-way Feedback	Physician Dietitian / No	No	36W

						activity	Email				

W, Week.

Multimedia Appendix 4 (continued)

Description of Internet-based self-monitoring interventions in 9 selected studies.

Author, year [Reference]	Components of self-monitoring intervention					Functionality	Facilities	Transmission / Interactivity	Provider / Peer support	Theory	Follow-up
	Glycaemic	Diet	Activity	Weight	Adherence						
Pérez-Ferre et al. 2010a,b (37, 38)	Y	Y	N	N	Y	Graphically progress	Website Phone SMS Glucometer	Asynchronous /Two-way Feedback	Physician (endo-crinologist) Nurse (nurse educator) / No	No	No
